# Evaluation of the use of *Idesia polycarpa* Maxim protein coating to extend the shelf life of European sweet cherries

**DOI:** 10.3389/fnut.2023.1283086

**Published:** 2023-11-17

**Authors:** Wenqing Yang, Zimu Zhang, Yaobing Chen, Kai Luo

**Affiliations:** College of Biological and Food Engineering, Hubei Minzu University, Enshi, China

**Keywords:** *Idesia polycarpa* Maxim, protein film, antioxidant activity, sweet cherry, shelf life

## Abstract

*Idesia polycarpa* Maxim protein was used as a substrate to prepare a novel food packaging material with bioactive functions for encapsulating and extending the postharvest shelf life of sweet cherries. The film-forming solution was prepared from a mixture of *Idesia polycarpa* Maxim protein, glycerol, and gelatin, and was cast to form a film at room temperature and evaluated for mechanical, optical, structural, crystallinity, thermal properties, morphology, and antioxidant activity. *Idesia polycarpa* Maxim protein composite film solution was applied as an edible coating on sweet cherries and evaluated for changes in physical and biochemical parameters of sweet cherries in storage at 20°C and 50% relative humidity for 9 days. The results showed that the film tensile strength increased from 0.589 to 1.981 Mpa and the elongation at break increased from 42.555% to 58.386% with the increase of *Idesia polycarpa* Maxim protein concentration. And in the *in vitro* antioxidant assay, IPPF-4.0% was found to have the best antioxidant activity, with scavenging rates of 65.11% ± 1.19%, 70.74% ± 0.12%, and 90.96% ± 0.49% for DPPH radicals, ABTS radicals, and hydroxyl radicals, respectively. *Idesia polycarpa* Maxim protein coating applied to sweet cherries and after storage at 20°C and 50% relative humidity for 9 days, it was found that the *Idesia polycarpa* Maxim protein coating significantly reduced the weight loss (54.82% and 34.91% in the Control and Coating-2.5% groups, respectively) and the loss of ascorbic acid content (16.47% and 37.14% in the Control and Coating-2.5% groups, respectively) of the sweet cherries, which can effectively extend the aging of sweet cherry fruits and prolong their shelf life. The developed protein film of *Idesia polycarpa* Maxim with antioxidant activity can be used as a new food packaging material in the food industry.

## 1 Introduction

Sweet cherries are produced in Western Europe and are the source species for most table cherries, their fruit is sweet and tart and can be eaten fresh or made into jams, wines, and canned goods. The bright red color of European sweet cherries is due to iron ions, but also to the richness of polyphenolic antioxidants such as anthocyanins, proanthocyanidins, lutein, and zeaxanthin, which fight cell damage, reduce inflammation, and act as antioxidants, anti-aging, prevent chronic diseases and promote overall health (1). European sweet cherries are a better choice for people with diabetes and hypertension due to their low sugar content, glycemic index of 22, low calorie content, and no sodium (2, 3). European sweet cherries are seasonal fruit, picked when ripe in May or June to obtain more nutrition, color, and flavor. Sweet cherries can be stored for 5–7 days and are severely damaged by microbial infestation, spoilage due to microbial multiplication, and water loss during growth, harvest, and storage (4–6). The use of food packaging to extend the shelf life of European sweet cherries is particularly critical.

At present, the preservation technology of sweet cherries mainly adopts the traditional low-temperature packaging method, which can effectively inhibit the growth of microorganisms to delay the aging and spoilage of sweet cherries (7). Although low-temperature packaging extends the shelf life of sweet cherries, but the cost is too high, and the collision between the fruits in the process of transportation will also cause some damage (8, 9). In recent years, the proposed edible active packaging method has effectively solved this problem (10). Edible packaging typically uses sustainable, biodegradable materials that are wrapped or coated around food products as consumables and do not generate waste (11). Edible coatings can be safely consumed and retain the taste and appearance of fresh fruit, and the coatings have some nutritional value (12). In addition, they should act as an effective semi-permeable barrier between the surrounding atmosphere and the fruit, blocking water vapor and respiratory gases (13). Biocoatings control physical and biochemical changes in the fruit during long-term storage by reducing the rate of respiration and metabolic reactions and by modifying the internal atmosphere (14). Not only are biopolymers biodegradable, but the raw materials are renewable resources (15). Natural edible polymers generally include polysaccharides, lipids, and proteins (16). Proteins are widely used for the production of biodegradable films due to their excellent film-forming ability. Compared to films made from polysaccharides and lipids, protein-based films have better physical, nutritional, and functional activities (17). For example, edible film or coating treatments preserved the post-harvest quality of sweet cherries by minimizing weight loss and ascorbic acid loss, inhibiting fungal growth, and maintaining the firmness of the coated fruit (18, 19). Milk protein concentrate-based coatings successfully delayed the darkening, shriveling, and softening of the cherries (20). The edible film coating acts as a barrier, which can inhibit gas exchange, reduce respiration rates, and slow down fruit decay (21). *Nigella sativa* protein concentrate films by using grape juice have been applied to preserve the freshness of sweet cherries (22). The protein-based film improved the antimicrobial and antioxidant properties of the film by adding grape juice to it. Fungal growth was inhibited and the shelf life of fresh sweet cherries was extended (23). Edible Carboxymethyl Chitosan-Gelatin based coating was beneficial in delaying the decay of sweet cherries. After 33 days of storage, the fruit decay rate of the coated group only ranged from 3.0% to 15.3%, while that of the control group reached 17.7%–63.0% (24). It was shown that the protein-based edible coating can reduce the respiration and transpiration rates of sweet cherries, minimize firmness loss, and reduce fruit decay (25). Yuan et al. (26) found that the use of multifunctional electrostatically spun nanofibre films loaded with protein-based biopolymers for packaging sweet cherries effectively delayed fruit senescence and maintained postharvest quality. However, due to the slow speed of nanopreparation by electrostatic spinning and the high economic cost of raw material preparation, it is not suitable for large-scale production and application. Therefore, it is crucial to find a biodegradable, sustainable, inexpensive packaging material with a simple preparation process to prolong the freshness of sweet cherries.

*Idesia polycarpa* cake meal is a by-product of *Idesia polycarpa* oil extraction in which *Idesia polycarpa* protein is the main nutrient. According to the preliminary experimental research in our laboratory, *Idesia polycarpa* fruit pomace is also rich in protein, and the preliminary *in vitro* antioxidant experiments have shown that *Idesia polycarpa* protein has excellent antioxidant functions. A composite film with antioxidant function was prepared by using *Idesia polycarpa* protein and the film was formed on the surface of European sweet cherry by a coating method. The mechanical properties, optical properties, and antioxidant activity of *Idesia polycarpa* protein composite films were also investigated. The biochemical properties of the *Idesia polycarpa* protein composite films were also studied for changes in the storage of European sweet cherries packaged with *Idesia polycarpa* protein composite films (9 d, 20°C), and the effect of *Idesia polycarpa* protein composite films on the shelf-life of European sweet cherries was investigated.

## 2 Materials and methods

### 2.1 Materials

*Idesia polycarpa* was sourced from Enshi, Hubei, China, and the *Idesia polycarpa* cake meal used in the study was sourced from the by-products of *Idesia polycarpa* oil extraction. Ripe and fresh sweet cherries were obtained from Spain. Hexane and sodium hydroxide were analytical reagent grade and were purchased from Tianjin Hengxing Chemical Reagent Manufacturing Co., Ltd. Gelatin was purchased from Aladdin Reagent Database Inc. (Shanghai, China). Glycerol was purchased from Taiko Palm-Oleo (Zhangjiagang) Co., Ltd.

### 2.2 Protein extraction

The protein was prepared according to the method of Das et al. (27) with some modifications. The *Idesia polycarpa* cake meal was crushed and passed through an 80 mesh sieve, then the *Idesia polycarpa* cake meal flour was defatted with n-hexane and finally air dried in a fume hood for 48 h to obtain defatted *Idesia polycarpa* cake meal flour. The defatted *Idesia polycarpa* cake meal flour was mixed with 0.06 mol/L NaOH solution in a material-liquid ratio of 1:55. The mixture was heated to 53°C using a magnetic stirrer (DF-101S, China) and then stirred for 2 h. The supernatant was collected by centrifugation (3,578×*g*, 10 min). The pH of the supernatant was adjusted to 3.2 with 1 mol/L HCl. After 2 h at room temperature, the precipitate was collected by centrifugation (3,578×*g*, 10 min), washed with distilled water to neutral, and dried in a freeze dryer (SCIENTZ-100F/A, China) for 24 h to obtain the *Idesia polycarpa* protein.

### 2.3 Film preparation

*Idesia polycarpa* protein (IPP) was dissolved in distilled water (2.5%, 3.0%, 3.5%, and 4%), the pH of the solution was adjusted to 12 with 2 mol/L NaOH, 30% (v/v) glycerol was added and stirred at 40°C for 1 h under magnetic stirring to mix well. The temperature was raised to 70°C and 0.25% (w/v) gelatin was added and stirred for 20 min. After the solution was cooled to room temperature, 8 mL of film-forming solution was evenly distributed on disposable Petri dishes (100 mm). The disposable Petri dishes were placed in an oven at 25°C for 24 h and then the films were removed (28). The composite films with concentrations of 2.5%, 3%, 3.5%, and 4% were recorded as IPPF-2.5%, IPPF-3.0%, IPPF-3.5% and IPPF-4.0%, respectively.

### 2.4 Film characterization

#### 2.4.1 Thickness

Film thickness was measured with a micrometer. The thickness of the film samples was determined by averaging six random measurements.

#### 2.4.2 Mechanical performance

The tensile strength (TS) and elongation at break (EAB) of the film were tested with the help of a computerized tensile tester (DR-508A, China). The film was cut into long strips of 10 mm × 60 mm, equilibrated at 25°C and 30% relative humidity for 24 h and fixed on the tensile tester, stretched at a rate of 15 mm/min with an initial length of 35 mm, and the tensile strength and elongation at break were calculated according to the following equations.


$$TS=\frac{F}{d\times b}$$

Where F is the maximum tensile force that the film can withstand, d is the thickness of the film, and b is the width of the film.


$$EAB=\frac{L-L_{0}}{L_{0}}\times 100\%$$

where L is the length of the film when it breaks and L_0_ is the initial length of the film.

#### 2.4.3 Moisture content (MC)

The moisture content of the film is tested by a moisture tester (HE53/02, China). 0.5–1 g of the film was placed in the moisture tester for testing.

#### 2.4.4 Water vapor permeability (WVP)

Water vapor permeability was measured according to the method of Zhang et al. (29) with some modifications. 10 mL of distilled water was added to a permeable cup with an inner diameter of 6 cm, and the permeable cup was covered with a 7 cm × 7 cm film sample. The change in weight of the distilled water in the permeable cup was recorded after 24 h at 25°C and 30% relative humidity.

#### 2.4.5 Color

The color parameters (L*, a*, and b*) of six random points of the film samples were measured using a colorimeter (CS-820 N, China). The total color difference (∆E) and the whiteness index (WI) of the film were calculated using the following equation.


$$\Delta E={\left[{\left(\Delta L^{*}\right)}^{2}+{\left(\Delta a^{*}\right)}^{2}+{\left(\Delta b^{*}\right)}^{2}\right]}^{1/2}$$

$$WI=100-{\left[{\left(100-L^{*}\right)}^{2}+{\left(a^{*}\right)}^{2}+{\left(b^{*}\right)}^{2}\right]}^{1/2}$$

#### 2.4.6 Opacity (OP)

The opacity of the film was measured using a UV spectrophotometer (UV-8000H, China). The film was cut into rectangles (1 cm × 5 cm) and the absorbance at 600 nm was measured by fixing the film on the surface of the cuvette and an empty cuvette was used as a blank control.


$$\mathrm{Opacity}=\frac{A_{600}}{t}$$

where A_600_ is the film sample absorbance at 600 nm and t is the film sample thickness (mm).

#### 2.4.7 Fourier transform infrared spectroscopy (FTIR)

FTIR spectra of the film were collected using an FTIR spectrometer (BRUKER, America) in the wave number range of 4,000–500 cm^−1^.

#### 2.4.8 X-ray diffraction (XRD)

The samples were scanned from 5° to 70° at 2°/min with Cu Kα source at 40 kV and 40 mA by X-ray diffractometer (Bruker D8 advance, Germany).

#### 2.4.9 Differential scanning calorimeter (DSC)

DSC measurements (Netzsch DSC214, Germany) were performed on the film samples using a differential scanning calorimeter. Nitrogen gas was flushed at a rate of 20 mL/min (as cooling gas). The film samples (5–10 mg) were sealed in an aluminum tray and heated from 30°C to 200°C at a rate of 10°C/min.

#### 2.4.10 Scanning electron microscope (SEM)

The morphology of the film sample was imaged by SEM (TESCAN MIRA LMS, Czech Republic), and the morphology was recorded with an accelerating voltage of 3 kV. The sample was fractured in liquid nitrogen and the fracture surface was observed after gold sputtering.

### 2.5 Anti-oxidation performance

The antioxidant activity of the *Idesia polycarpa* protein film was determined by immersing 100 mg of film samples in 10 mL of distilled water and stirring until complete dissolution.

#### 2.5.1 DPPH

Referring to the method of Maryam Adilah and Nur Hanani (30) with slight modifications. DPPH solution (0.1 mmol/L) was prepared in ethanol. 1 mL of film sample solution was mixed well with 3 mL of DPPH solution and the reaction was carried out at room temperature for 30 min protected from light, and the absorbance was measured at 517 nm as A_1_. The sample control group was substituted with distilled water for the DPPH solution and the absorbance value was measured as A_2_; the blank group was substituted with distilled water for the sample solution and the absorbance value was measured as A_0_; and the DPPH scavenging activity was obtained using the following equation.


$$\mathrm{DPPH}\;\mathrm{radical}\;\mathrm{scavenging}\;\mathrm{activity}\left(\%\right)=\left(1-\frac{A_{1}-A_{2}}{A_{0}}\right)\times 100$$

#### 2.5.2 ABTS

According to the method of Moghadam et al. (31) with slight modifications. The aqueous ABTS solution (7 mmol/L) was mixed with an equal volume of potassium persulfate solution (2.45 mmol/L), placed in a refrigerator at 4°C for 16 h, and diluted with distilled water to an absorbance of 0.7 ± 0.02 at use. 0.2 mL of film sample solution was added to 4 mL of ABTS dilution solution, protected from light, and reacted for 6 min, and the absorbance value at 734 nm was measured as A_1_; the sample control group was treated with distilled water instead of ABTS solution and the absorbance was measured as A_2_; the blank group was measured as A_0_ with distilled water instead of film sample solution, and the ABTS scavenging activity was obtained using the following equation.


$$\mathrm{ABTS}\;\mathrm{radical}\;\mathrm{scavenging}\;\mathrm{activity}\left(\%\right)=\left(1-\frac{A_{1}-A_{2}}{A_{0}}\right)\times 100$$

#### 2.5.3 Hydroxyl radicals

The method of Zhang et al. (32) was used with slight modifications. 1 mL of FeSO_4_ solution with a concentration of 6 mmol/L, 1 mL of salicylic acid solution with a concentration of 6 mmol/L, and 1 mL of sample solution were added to the test tube. After mixing, 1 mL of H_2_O_2_ solution with a concentration of 6 mmol/L was added. After mixing again, the reaction was carried out at 37°C for 30 min and the absorbance of the solution was measured at 510 nm as A_1_. The absorbance was measured as A_2_ for the sample control group using distilled water instead of H_2_O_2_ solution and as A_0_ for the blank group using distilled water instead of sample solution. The hydroxyl radical scavenging capacity was calculated as follows.


$$\mathrm{Hydroxyl}\;\mathrm{radical}\;\mathrm{scavenging}\;\mathrm{activity}\left(\%\right)=\left(1-\frac{A_{1}-A_{2}}{A_{0}}\right)\times 100$$

### 2.6 Application of film in sweet cherry

#### 2.6.1 Sample preparation

Sweet cherries were coated with reference to the method of Basiak et al. (33). Sweet cherries of uniform size and without mechanical damage were selected for experimental work. The sweet cherries were soaked in *Idesia polycarpa* protein film solution for 1 min and air-dried intermittently for 10 min, with three soaking times. The coating was formed by allowing the film solution on the surface of the sweet cherries to air dry. The effect of two different concentrations (2.5% and 4%) of *Idesia polycarpa* protein film solution on sweet cherries was evaluated and recorded as Coating-2.5% and Coating-4.0%. The untreated sweet cherries were recorded as the control group as Control. All fruits were placed in storage at 20°C and 50% humidity.

#### 2.6.2 Weight loss

The weights of the control and coated sweet cherries were recorded daily. Differential weight loss was calculated and converted to percentages.

#### 2.6.3 pH and titratable acidity (TA)

5 g of sweet cherry pulp was homogenized in distilled water (50 mL) and filtered. The pH of the filtrate was measured with a pH meter.

The TA of the samples was evaluated according to the method of Colussi et al. (34) with slight modifications. 5 g of the samples were stirred in distilled water (50 mL) at 80°C for 2 h and filtered. Then 20 mL of the filtrate was titrated with 0.1 mol/L NaOH solution to pH 8.1. TA was expressed as grams of citric acid per 100 g of sample weight and was determined as follows


$$TA=\left[V\left(\mathrm{NaOH}\right)\times 0.1\times 0.064/m\right]\times 100\%$$

where V(NaOH) is the volume of titrated NaOH(mL), 0.1 is the molar concentration of NaOH solution, 0.064 is the conversion factor of citric acid, and m(g) is the mass of the aliquot.

#### 2.6.4 Total soluble sugars (TSS)

Total soluble sugar content was determined according to the method of Gharibzahedi et al. (35) with some modifications. 5 g of sweet cherry pulp was pounded in distilled water (50 mL), stirred for 2 h at 80°C, and filtered. 1 mL of filtrate, 1 mL of phenol solution (5%), and 5 mL of H_2_SO_4_ were taken in a test tube and mixed well and put the test tube into a 90°C water bath for 30 min reactions, and then the absorbance was measured at 490 nm using a UV spectrophotometer with distilled water as a blank. The regression equation used was y = 0.0086 x + 0.1412 (R^2^ = 0.9987).

#### 2.6.5 Ascorbic acid

The content of ascorbic acid was enumerated by using 2,6-dichlorophenol indophenol (DCPIP) dye (36). Sweet cherry pulp (1 g) was mashed in 10 mL of HPO_3_ (2%) and mixed with gentle shaking in the dark for 30 min. The supernatant was collected by centrifugation (3,578×*g*, 10 min). The supernatant (350 μL) was diluted 1:10 times with 2% HPO_3_ and mixed with DCPIP dye (650 μL), incubated in the dark for 3 min and the absorbance was measured at 518 nm with 80% ethanol as a blank. The regression equation used was y = −0.0946 x + 1.434 (R^2^ = 0.9966) and was correlated with absorbance (x) and ascorbic acid concentration (y).

### 2.7 Statistical analysis

All experiments were performed in triplicate (unless otherwise stated), and data in the text are reported as mean ± standard deviation and statistically evaluated using the OriginPro 2023 one-way analysis of variance (ANOVA) test and accepted as statistically significant at *p* < 0.05.

## 3 Results and discussion

### 3.1 Physicochemical properties of the protein film of *Idesia polycarpa*

Mechanical properties are one of the criteria for assessing whether a film can be used as a food packaging material, and the film or coating material can act as a protective layer for the food, reducing the risk of mechanical damage to the food. The physicochemical properties of *Idesia polycarpa* protein films are shown in Table 1, where we can find that the thickness, TS, and EAB of the films increased with the increase in protein concentration. Also with increasing protein concentration WVP decreased from 9.26% ± 0.09% to 8.44% ± 0.09%, which indicates that the increase in protein concentration favors the decrease in WVP of the films. This could be because the tighter structures formed by the larger concentration of protein solutions during film formation block the passage of water molecules, which is consistent with the results reported by Bishnoi et al. (37) in wheat protein-alginate composite films. This result is also supported by the detection of film moisture content, which increased with increasing protein concentration, with 2.367% for IPPF-2.5% and 4.950% for IPPF-4.0%. As the protein concentration increases the film structure becomes more compact resulting in a slower evaporation of moisture, which is beneficial for applications in the food packaging industry.

**Table 1:** Thickness, tensile strength, elongation at break, moisture content, and water vapor permeability of films.

Film type	Thickness (mm)	TS (Mpa)	EAB (%)	MC (%)	WVP (g/cm^3^·day)
IPPF-2.5%	0.196 ± 0.011^d^	0.589 ± 0.064^d^	42.555 ± 2.493^c^	2.367 ± 0.233^c^	9.26% ± 0.09%^c^
IPPF-3.0%	0.227 ± 0.011^c^	0.978 ± 0.080^c^	43.817 ± 1.779^c^	2.873 ± 0.137^c^	9.09% ± 0.03%^b^
IPPF-3.5%	0.246 ± 0.017^b^	1.472 ± 0.053^b^	49.353 ± 2.285^b^	4.057 ± 0.189^b^	8.74% ± 0.06%^a^
IPPF-4.0%	0.296 ± 0.007^a^	1.981 ± 0.042^a^	58.386 ± 2.051^a^	4.950 ± 0.118^a^	8.44% ± 0.09%^a^

Different lowercase letters in the same column indicates significant differences (*p* < 0.05).

### 3.2 Optical properties of the protein film of *Idesia polycarpa*

The overall brown color of the film was mainly due to the richness of natural pigments in *Idesia polycarpa*. In contrast, the *Idesia polycarpa* cake meal was not decolorized during the protein extraction process. The results of the optical properties of the films are shown in Table 2. The total color difference and whiteness index of the films showed a decreasing trend with increasing protein concentration. While opacity is positively correlated with protein concentration, this is because the introduction of pigment increases with protein concentration. The lightest brown color L* was 29.278 ± 0.365, a* was 4.210 ± 0.185, and b* was 6.645 ± 0.482 for the IPPF-2.5% films. The darkest brown color L* was 26.452 ± 0.116, a* was 3.028 ± 0.119, and b* was 2.455 ± 0.150 for the IPPF-4.0% films. This is because the natural color of *Idesia polycarpa* proteins is brown, so the color of the film will gradually darken with increasing protein content. This brown shadeable film and the film with high opacity are suitable for storing photosensitive foods (38). For some photosensitive foods, such films can block specific wavelengths of light and can effectively prevent the coated foods from oxidation reactions (39, 40).

**Table 2:** Film color properties and opacity.

Film type	L*	a*	b*	∆E	WI	OP
IPPF-2.5%	29.278 ± 0.365	4.210 ± 0.185	6.645 ± 0.482	30.321 ± 0.396	28.840 ± 0.354	1.711 ± 0.034
IPPF-3.0%	28.075 ± 0.189	4.122 ± 0.121	5.208 ± 0.512	28.854 ± 0.262	27.767 ± 0.167	2.538 ± 0.027
IPPF-3.5%	27.290 ± 0.177	3.722 ± 0.094	3.492 ± 0.290	27.765 ± 0.147	27.111 ± 0.189	3.065 ± 0.037
IPPF-4.0%	26.452 ± 0.116	3.028 ± 0.119	2.455 ± 0.150	26.738 ± 0.128	26.348 ± 0.112	3.379 ± 0.037

### 3.3 FTIR

**Figure 1:**
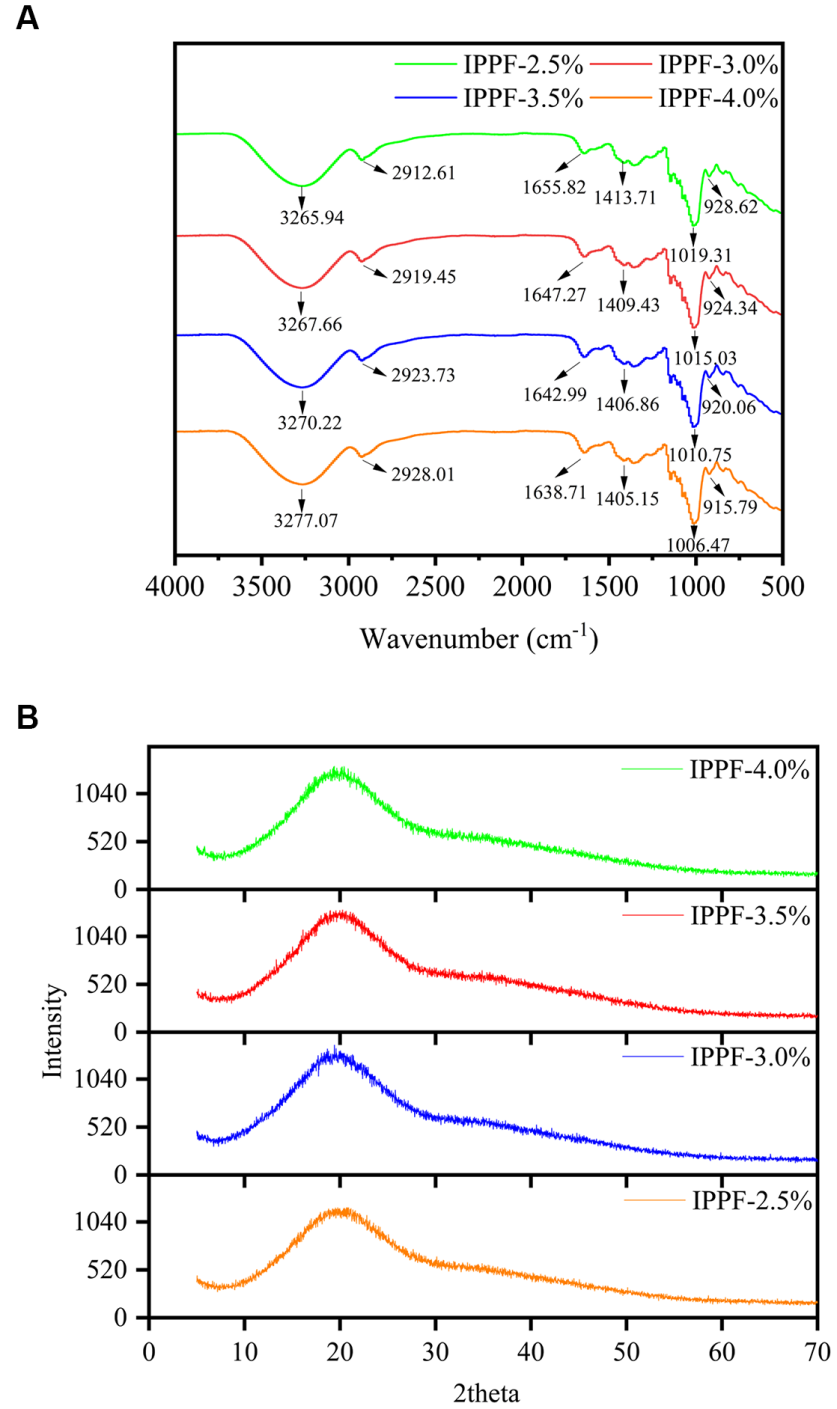
FTIR spectra **(A)** and X-ray diffractograms patterns **(B)** of IPPF-2.5%, IPPF-3.0%, IPPF-3.5%, and IPPF-4.0% films.

The FTIR spectra of the films prepared from four different concentrations of *Idesia polycarpa* proteins are shown in Figure 1A, from which it can be clearly observed that the curves of the films with four different concentrations of *Idesia polycarpa* proteins are basically the same. Significant protein characteristic peaks can also be observed, with absorption bands at 1,653 cm^−1^ and 1,530 cm^−1^ for protein amide I and amide II bands, respectively (41). 1,447 cm^−1^ is the absorption peak for C-H stretching vibration and N-H bending vibration (amide III) (42). The FTIR spectra of CH at 3,253 cm^−1^ shows typical absorption bands associated with -OH groups and -NH_2_ groups associated with typical absorption bands (43). The absorption peak at 2,930 cm^−1^ is mainly the asymmetric stretching of the C-H bond in the CH_3_ group, and the absorption peak at 1,022 cm^−1^ is mainly the absorption peak of -C-O-C- in the glycosidic bond (44, 45). This phenomenon was attributed to the addition of gelatin to the membrane solution during the fabrication of the *Idesia polycarpa* protein membrane to obtain better mechanical properties, so the characteristic peaks of polysaccharides appeared in the infrared spectra of the films (46). According to Figure 1A, the four different concentrations of *Idesia polycarpa* protein complex membranes showed significant absorption peaks between 1,600 cm^−1^ and 1,700 cm^−1^, which is a region commonly used to study the secondary structure of amide I (47). The four different concentrations of *Idesia polycarpa* protein complex membranes showed obvious absorption peaks at 1,650 cm^−1^, which is mainly due to the strength of hydrogen bond between C=O and N-H bonds, indicating that *Idesia polycarpa* protein is mainly α-helical structure (48).

### 3.4 XRD

Figure 1B shows the X-ray diffraction patterns of the films prepared from different concentrations of *Idesia polycarpa* protein. For the thin film samples with various protein concentrations, it is evident from Figure 1B that characteristic peaks are visible around 2θ = 20°, which we analyzed to be primarily induced by the amorphous phase of the proteins, showing that the majority of the structures in all samples are amorphous. This is consistent with the results of Mir et al. (49) and Sarıcaoğlu and Turhan (50). Therefore, the XRD results showed that the *Idesia polycarpa* protein and glycerol were well-compatible in the preparation of edible membranes.

### 3.5 DSC

**Figure 2:**
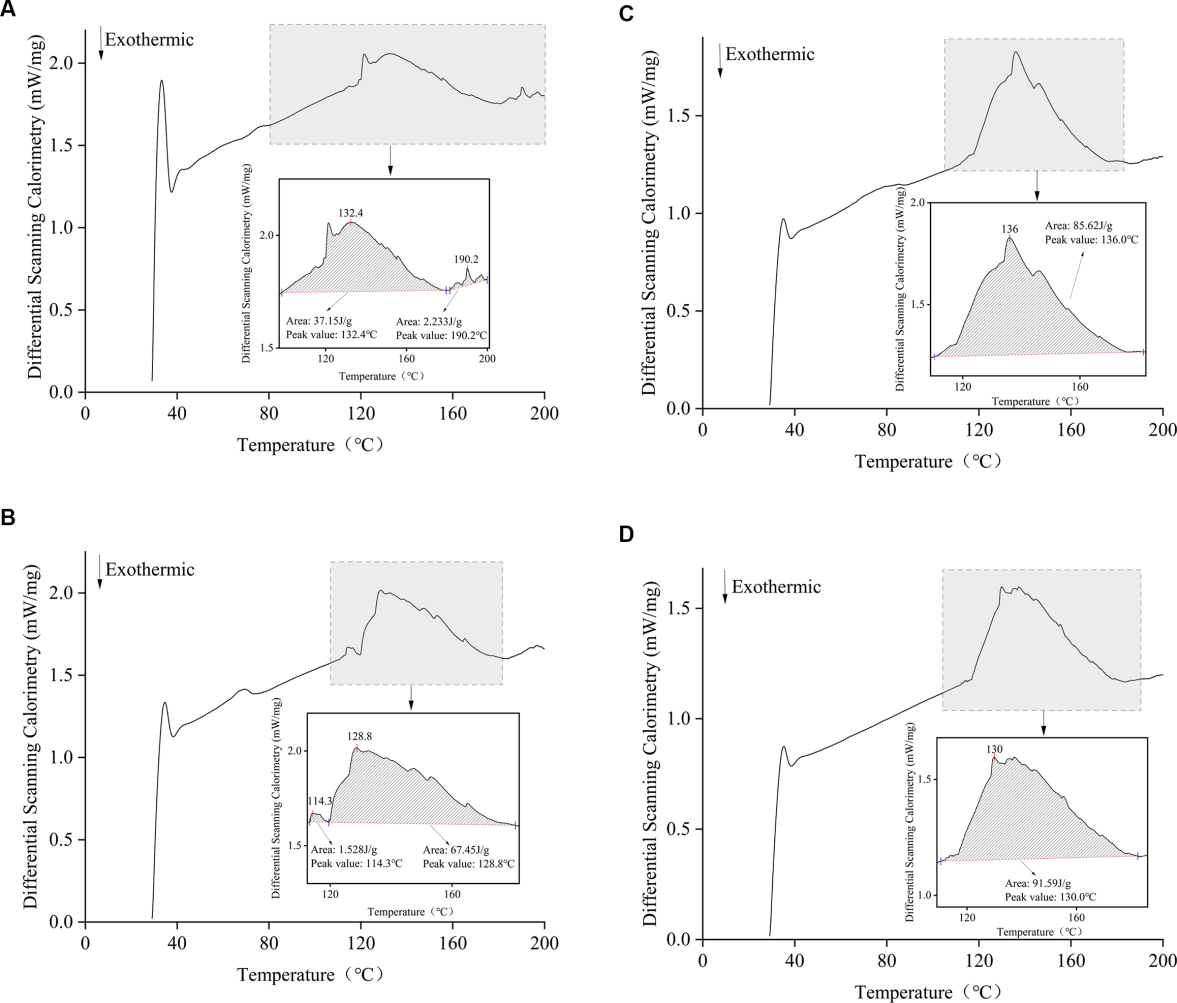
Differential scanning calorimetric curves for IPPF-2.5% **(A)**, IPPF-3.0% **(B)**, IPPF-3.5% **(C)**, and IPPF-4.0% **(D)** films.

A differential scanning calorimeter measures the change in the heat flow rate of a sample. The relationship between the energy difference between the substance and the reference during the heating process and the temperature is measured. The DSC thermograms of the films treated with different *Idesia polycarpa* protein contents are shown in Figure 2, with a clear exothermic peak observed at 110–185°C, which is caused by the thermal degradation of the films (51). The heat flow rate of the films decreased with the increase of *Idesia polycarpa* protein concentration. The peak areas of 37.15 J/g, 67.45 J/g, 85.62 J/g, and 91.59 J/g for IPPF-2.5%, IPPF-3.0%, IPPF-3.5%, and IPPF-4.0% films, respectively, were positively correlated with the *Idesia polycarpa* protein concentration, which may be due to the higher hydrophilicity of *Idesia polycarpa* protein after denaturation (52). This resulted in a higher water binding capacity of the *Idesia polycarpa* protein film, which is consistent with the results of water content measurement.

### 3.6 SEM of the protein film of *Idesia polycarpa*

**Figure 3:**
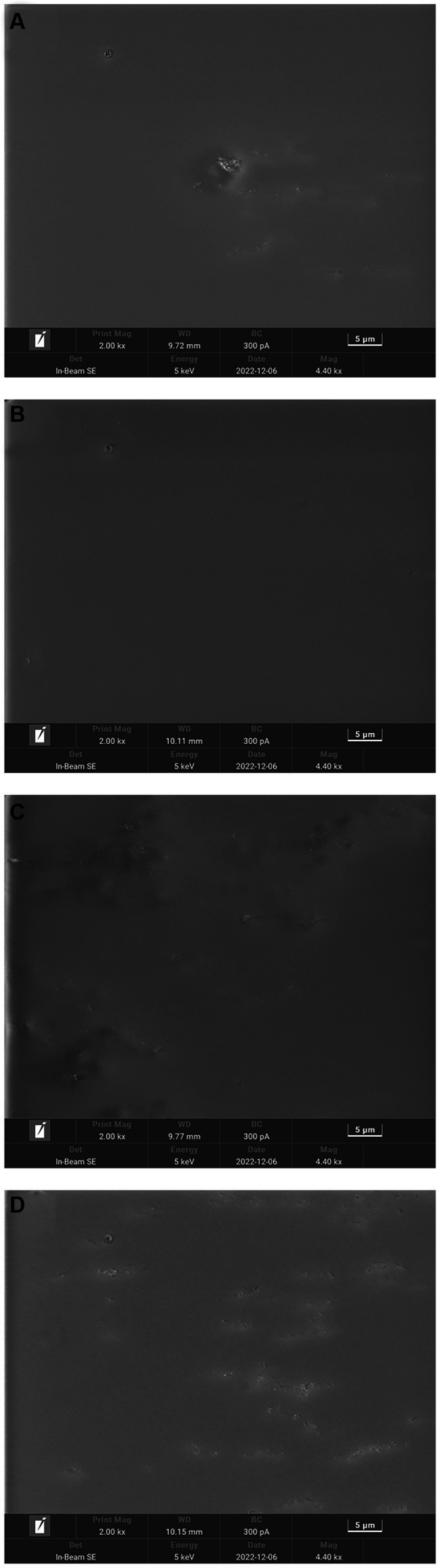
Scanning electron micrographs of IPPF-2.5% **(A)**, IPPF-3.0% **(B)**, IPPF-3.5% **(C)**, and IPPF-4.0% **(D)** film samples.

Scanning electron microscopy (SEM) was used to reveal the morphological properties, internal structure, and interactions among the constituents of the edible films. SEM images of the films containing different concentrations of *Idesia polycarpa* proteins are shown in Figure 3. It is obvious from Figure 3 that the IPPF-2.5% film and IPPF-3.0% film are smoother and more uniform, which indicates that the protein can be well integrated with glycerol. While IPPF-3.5% and IPPF-4.0% films were relatively rough and appeared fine particles, which was related to the solubility of the protein. As the protein concentration increased, the viscosity of the film-forming solution also increased, and the protein became increasingly difficult to dissolve. The appearance of fine particles may be due to incomplete dissolution. A similar situation was found in the films prepared from Fenugreek Protein (53).

### 3.7 Antioxidant activity

**Figure 4:**
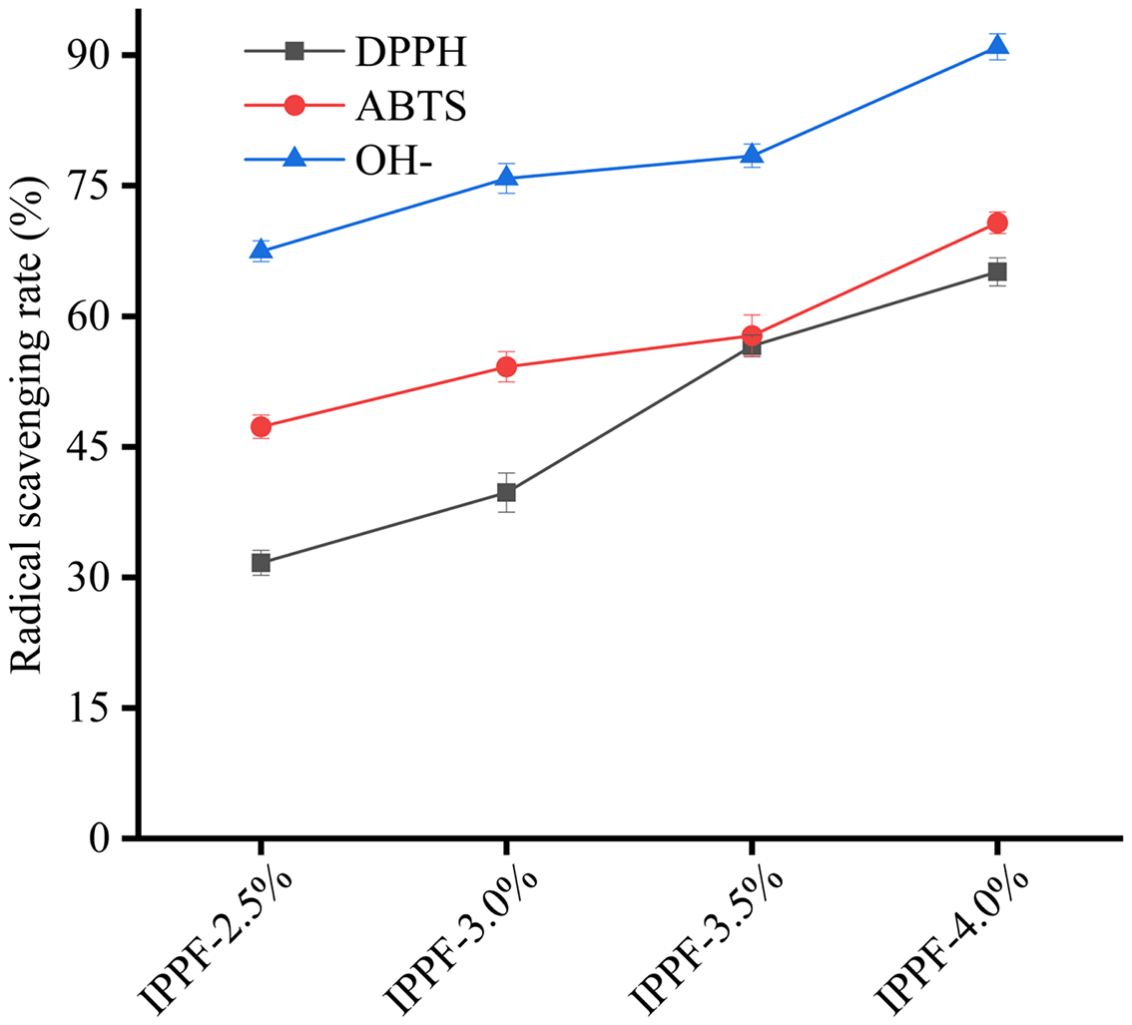
DPPH, ABTS, and hydroxyl radical scavenging capacity of IPPF-2.5%, IPPF-3.0%, IPPF-3.5%, and IPPF-4.0% films.

Antioxidant packaging is one of the main types of active packaging that can extend the shelf life of food products. Figure 4 shows that *Idesia polycarpa* protein films have strong antioxidant activity. When the concentration was 10 mg/mL, the DPPH radical scavenging rate was 31.67% ± 0.24%, 39.74% ± 0.67%, 50.59% ± 0.23%, and 65.11% ± 1.19% for IPPF-2.5%, IPPF-3.0%, IPPF-3.5%, and IPPF-4.0%, respectively, and the ABTS radical scavenging rate was 47.31% ± 0.52%, 54.20% ± 0.83%, 57.79% ± 0.74% and 70.74% ± 0.12%, and hydroxyl radical scavenging rates were 67.47% ± 0.23%, 75.85% ± 0.41%, 78.44% ± 0.25%, and 90.96% ± 0.49%, respectively. The antioxidant activity of the film depends on the content of the *Idesia polycarpa* protein. The higher the protein concentration, the higher the antioxidant activity of the protein film. The protein content is positively correlated with the antioxidant activity of the protein film. According to the results of the team’s previous research, *Idesia polycarpa* protein has good antioxidant function and its hydrolyzed products are mainly aspartic acid and glutamic acid, which have good antioxidant activity (54). For example, Lu et al. (55) found that sesame protein hydrolysis products are mainly composed of aspartic acid and glutamic acid, which is associated with their significant antioxidant activity. This result is similar to the results of this paper. This phenomenon was attributed to the good antioxidant property of *Idesia polycarpa* protein membrane is that *Idesia polycarpa* protein is insoluble in water under neutral conditions and soluble in water under alkaline conditions. Hydrolysis of *Idesia polycarpa* protein under alkaline conditions produces aspartic acid and glutamic acid, two amino acids that promote peroxidase activity or directly scavenge free radicals (56). This leads to good antioxidant activity of the *Idesia polycarpa* protein membrane. When it is used in functional packaging materials, it can effectively slow down the rate of oxidative aging of packaging products.

### 3.8 Preservation experiment of sweet cherry samples

#### 3.8.1 Morphological changes of sweet cherries during the 9 d storage period

**Figure 5:**
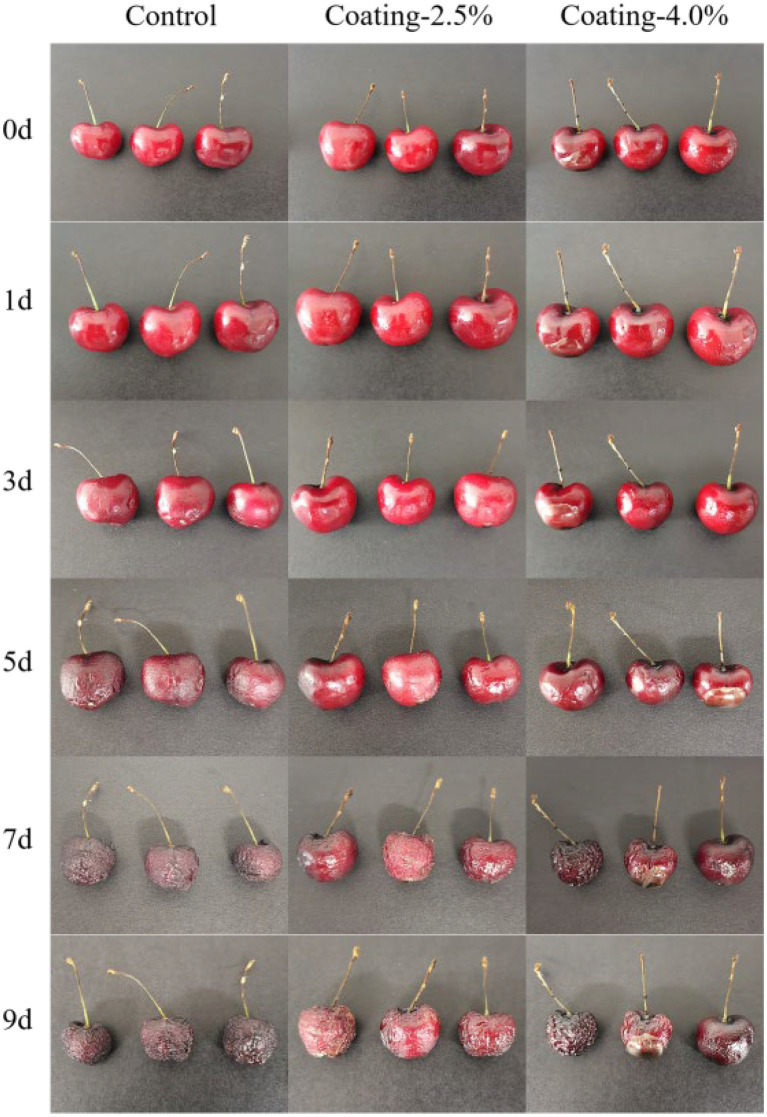
Changes in the appearance of sweet cherries untreated (Control), coated with 2.5% protein film solution (Coating-2.5%), and coated with 4.0% protein film solution (Coating-4.0%) during 9 days of storage at 20°C and 50% relative humidity.

The appearance and color of the fruit is an important fruit quality parameter that affects its marketability and consumer acceptability. In general, the shade of color represents the maturity of the fruit. During the whole storage period, the appearance of the fruit changed as shown in Figure 5. On day 5, it was observed that the sweet cherries in the Control group turned dark red in appearance, while the sweet cherries in the coated group had a bright red surface. The sweet cherries in the Control group aged faster than those in the coating group, and those in the Coating-2.5% group aged the slowest. The viscosity of the Coating suspension was critical in the dip coating process of the sweet cherries, and the Coating-4.0% suspension was very viscous, which prevented the dip coating process from achieving uniform film thickness, which would explain the fact that the Coating-2.5% group had a more uniform film thickness compared to the Coating-4.0% group has a better preservation function compared to the Coating-4.0% group.

#### 3.8.2 Weight loss

**Figure 6:**
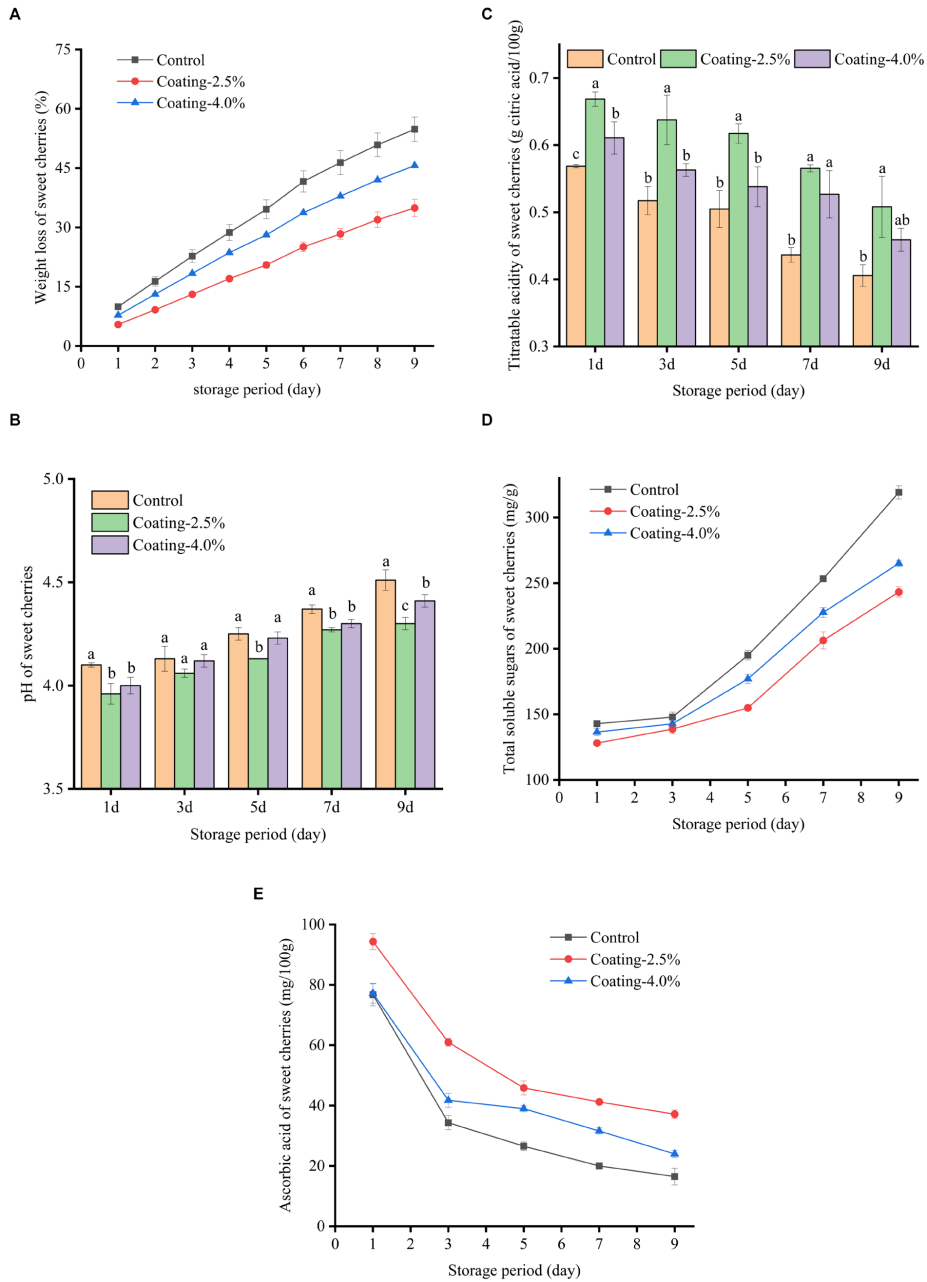
Changes in weight loss **(A)**, pH **(B)**, Titratable acidity **(C)**, Total soluble sugars **(D)**, and Ascorbic acid **(E)** content of untreated (Control), coated with 2.5% protein film solution (Coating-2.5%) and coated with 4.0% protein film solution (Coating-4.0%) of sweet cherries during 9 days of storage at 20°C and 50% relative humidity.

The weight loss of sweet cherries in different treatment groups during storage is shown in Figure 6A, where all sweet cherry samples showed continuous weight loss during storage. At the end of the storage period (9 d), the weight loss was 54.82%, 34.91%, and 45.64% for the Control, Coating-2.5%, and Coating-4.0% coating groups, respectively. The Coating-2.5% coating treatment significantly retarded the weight loss of sweet cherries compared to the Control (*p* < 0.05). Weight loss of fruits during storage is mainly due to water loss due to respiration and transpiration of fruits (57). The barrier formed by the edible coating on the fruit surface between the fruit and the external environment was able to reduce gas exchange and water loss, which was closely related to the lower water vapor permeability of the *Idesia polycarpa* protein composite membrane (58). At the same time, we found that the WVP of the *Idesia polycarpa* protein composite film with a concentration of 4.0% was lower than that of the *Idesia polycarpa* protein composite film with a concentration of 2.5% in the process of film manufacture by plate spreading. We have analyzed that the main reason for this opposite phenomenon is due to the different ways of spreading the film. When the film is spread on a flat plate, the film liquid does not flow in the plate, so the viscosity of the film liquid has less influence on the film-forming effect. While the infiltration film deposition method was used in the study of weight loss of sweet cherries during storage, in this method of film deposition, due to gravity affecting the flow of film liquid on the surface of sweet cherries, and the concentration of 4.0% *Idesia polycarpa* protein composite film liquid was more viscous, and the film deposition was not uniform. This resulted in greater weight loss in the 4.0% coating group.

#### 3.8.3 pH and TA

The pH, the titratable acidity of the fruit is an important quality parameter that reflects the ripeness of the fruit. The main organic acids found in sweet cherries are malic and citric acid, which are suitable for the acidity, taste, and flavor of the fruit. In this study, it was observed that regardless of the treatment done on sweet cherries, the pH values of sweet cherries were significantly higher and TA values (Figures 6B,C) were significantly lower (*p* < 0.05) throughout the storage period (9 d). At the end of the storage period at 20°C and 50% RH, the sweet cherries in the Control group had the highest pH (4.5), while the sweet cherries in the Coating-2.5% group had the lowest pH (4.3). This phenomenon can be explained by the fact that the high respiration rate of sweet cherries after harvesting converts starch and acid to sugar during storage, leading to an increase in sweet cherry pH (59). In contrast, the excellent barrier properties of the fruit coating reduced the amount of oxygen entering the fruit, effectively reducing the respiration rate, enzyme activity, and organic acid consumption of sweet cherries and delaying the decrease in acidity (60). TA values of sweet cherry samples increased significantly with pH (*p* < 0.05). The lowest TA value (0.41%) was observed in the control sweet cherries after 9 d of storage at 20°C and 50% RH. At this time, sweet cherry TA values in the Coating-2.5% and Coating-4.0% groups were 0.51% and 0.46%, respectively. As the sweet cherry fruit ripened and decayed, organic acids gradually decomposed into sugars, leading to a decrease in TA.

#### 3.8.4 TSS

TSS is a parameter that measures fruit senescence, and the increase in respiration during senescence increases the TSS of the fruit, which means that the increase in TSS can be used as a proxy for the senescence process. The initial metabolic process in fruits converts carbohydrates into sugars and other soluble compounds, leading to the accumulation of TSS during storage (61). As shown in Figure 6D, the content of total soluble sugars in sweet cherries increased with storage time during storage at 20°C and 9 d as a result of hydrolysis of starch and other carbohydrates in sweet cherries to produce monosaccharides (e.g., sucrose, glucose, fructose, etc.). During 9 days of storage, the TSS content was 319.19 ± 5.00, 243.23 ± 3.94, and 264.97 ± 2.45 mg/g for Control, Coating-2.5% and Coating-4.0%, respectively. The key reason for this phenomenon was the barrier effect of the coating, resulting in its lower metabolic rate and starch hydrolysis rate processes.

#### 3.8.5 Ascorbic acid

Ascorbic acid is a water-soluble vitamin that decreases with the evaporation of water during storage. The different treatment groups had a significant effect (*p* < 0.05) on the change of ascorbic acid content (Figure 6E) in sweet cherries. The results showed that the ascorbic acid content of the Control, Coating-2.5%, and Coating-4.0% groups were 16.47%, 37.14%, and 23.96%, respectively, after 9 days of storage period. This was attributed to the effectiveness of coating in reducing the rate of fruit respiration, enzyme activity, and evaporation of water vapor, thus reducing the loss of ascorbic acid content (62). Meanwhile, Yousuf et al. (63) made similar findings in coating experiments on fresh-cut melons using fruit extracts and hydrocolloids. This finding can effectively delay the rate of fruit deterioration and increase the commercial value of the fruit.

## 4 Conclusion

The feasibility of using *Idesia polycarpa* Maxim protein as a substrate for the preparation of edible films was demonstrated by the solution casting method. *Idesia polycarpa* Maxim protein composite film with good mechanical properties, barrier properties, and antioxidant activity. After storing sweet cherries at 20°C and 50% relative humidity for 9 days, the *Idesia polycarpa* Maxim protein-based bioactive coatings were found to exert beneficial effects on the shelf-life parameters of sweet cherries, with the best preservation results in the Coating-2.5% group. Extend the shelf life of sweet cherries by maintaining their color appearance, reducing weight loss, and decreasing the loss of ascorbic acid content. In addition, despite the high antioxidant activity of the designed films, Coating-2.5% showed superior freshness preservation than Coating-4.0% when it was applied to sweet cherries. This is related to the manner of coating, which is susceptible to the effect of solution viscosity when coating is performed by infiltration, resulting in non-uniform coating. However, all the coated groups showed better freshness retention than the control group. Therefore, *Idesia polycarpa* Maxim protein composite film is a good biofunctional food packaging material, which is also beneficial for direct use as fruit food packaging, which will greatly exploit the value of the utilization of *Idesia polycarpa* by-products.

## Data availability statement

The original contributions presented in the study are included in the article/supplementary materials, further inquiries can be directed to the corresponding author.

## Author contributions

WY: Conceptualization, Investigation, Writing – review & editing, Data curation, Methodology, Software, Validation, Visualization, Writing – original draft. ZZ: Conceptualization, Data curation, Investigation, Methodology, Validation, Visualization, Writing – original draft, Writing – review & editing, Supervision. YC: Investigation, Supervision, Writing – review & editing, Formal analysis, Project administration, Resources. KL: Formal analysis, Investigation, Project administration, Resources, Supervision, Conceptualization, Funding acquisition, Writing – review & editing.

## References

[1] Chockchaisawasdee S Golding JB Vuong QV Papoutsis K Stathopoulos CE. Sweet cherry: composition, postharvest preservation, processing and trends for its future use. Trends Food Sci Technol. (2016) 55:72–83. doi: 10.1016/j.tifs.2016.07.002

[2] Papapetros S Louppis A Kosma I Kontakos S Badeka A Papastephanou C. Physicochemical, spectroscopic and chromatographic analyses in combination with chemometrics for the discrimination of four sweet cherry cultivars grown in northern Greece. Foods. (2019) 8:442. doi: 10.3390/foods8100442, PMID: 31561515 PMC6835477

[3] Ricardo-Rodrigues S Laranjo M Agulheiro-Santos AC. Methods for quality evaluation of sweet cherry. J Sci Food Agric. (2023) 103:463–78. doi: 10.1002/jsfa.12144, PMID: 35870155

[4] Aglar E Ozturk B Guler SK Karakaya O Uzun S Saracoglu O. Effect of modified atmosphere packaging and ‘parka’ treatments on fruit quality characteristics of sweet cherry fruits (*Prunus avium* L. ‘0900 Ziraat’) during cold storage and shelf life. Sci Hortic. (2017) 222:162–8. doi: 10.1016/j.scienta.2017.05.024

[5] Zehra A Wani SM Bhat TA Jan N Hussain SZ Naik HR. Preparation of a biodegradable chitosan packaging film based on zinc oxide, calcium chloride, nano clay and poly ethylene glycol incorporated with thyme oil for shelf-life prolongation of sweet cherry. Int J Biol Macromol. (2022) 217:572–82. doi: 10.1016/j.ijbiomac.2022.07.013, PMID: 35810854

[6] Zhang C Gong H Liu Y. Effects of postharvest coating using chitosan combined with natamycin on physicochemical and microbial properties of sweet cherry during cold storage. Int J Biol Macromol. (2022) 214:1–9. doi: 10.1016/j.ijbiomac.2022.06.057, PMID: 35705124

[7] Correia S Schouten R Silva AP Gonçalves B. Factors affecting quality and health promoting compounds during growth and postharvest life of sweet cherry (*Prunus avium* L.). Front Plant Sci. (2017) 8:2166. doi: 10.3389/fpls.2017.02166, PMID: 29312407 PMC5742238

[8] Remón S Ferrer A Marquina P Burgos J Oria R. Use of modified atmospheres to prolong the postharvest life of Burlat cherries at two different degrees of ripeness. J Sci Food Agric. (2000) 80:1545–52. doi: 10.1002/1097-0010(200008)80:10<1545::AID-JSFA680>3.0.CO;2-X

[9] Trajkovska Petkoska A Daniloski D D’Cunha NM Naumovski N Broach AT. Edible packaging: sustainable solutions and novel trends in food packaging. Food Res Int. (2021) 140:109981. doi: 10.1016/j.foodres.2020.109981 33648216

[10] Nunes C Silva M Farinha D Sales H Pontes R Nunes J. Edible coatings and future trends in active food packaging–fruits’ and traditional sausages’ shelf life increasing. Foods. (2023) 12:3308. doi: 10.3390/foods12173308, PMID: 37685240 PMC10486622

[11] Xie Q Liu G Zhang Y. Edible films/coatings containing bioactive ingredients with micro/nano encapsulation: a comprehensive review of their fabrications, formulas, multifunctionality and applications in food packaging. Crit Rev Food Sci Nutr. (2022) 12:1–38. doi: 10.1080/10408398.2022.2153794 36503369

[12] Chavan P Lata K Kaur T Rezek Jambrak A Sharma S Roy S. Recent advances in the preservation of postharvest fruits using edible films and coatings: a comprehensive review. Food Chem. (2023) 418:135916. doi: 10.1016/j.foodchem.2023.135916, PMID: 37001356

[13] Maringgal B Hashim N Mohamed Amin Tawakkal IS Muda Mohamed MT. Recent advance in edible coating and its effect on fresh/fresh-cut fruits quality. Trends Food Sci Technol. (2020) 96:253–67. doi: 10.1016/j.tifs.2019.12.024

[14] Romanazzi G Moumni M. Chitosan and other edible coatings to extend shelf life, manage postharvest decay, and reduce loss and waste of fresh fruits and vegetables. Curr Opin Biotechnol. (2022) 78:102834. doi: 10.1016/j.copbio.2022.102834 36343563

[15] Perera KY Jaiswal AK Jaiswal S. Biopolymer-based sustainable food packaging materials: challenges, solutions, and applications. Foods. (2023) 12:2422. doi: 10.3390/foods12122422, PMID: 37372632 PMC10297947

[16] Mohamed SAA El-Sakhawy M El-Sakhawy MA-M. Polysaccharides, protein and lipid-based natural edible films in food packaging: a review. Carbohydr Polym. (2020) 238:116178. doi: 10.1016/j.carbpol.2020.116178, PMID: 32299560

[17] Iversen LJL Rovina K Vonnie JM Matanjun P Erna KH ‘Aqilah NMN. The emergence of edible and food-application coatings for food packaging: a review. Molecules. (2022) 27:5604. doi: 10.3390/molecules27175604, PMID: 36080371 PMC9457879

[18] Hassan B Chatha SAS Hussain AI Zia KM Akhtar N. Recent advances on polysaccharides, lipids and protein based edible films and coatings: a review. Int J Biol Macromol. (2018) 109:1095–107. doi: 10.1016/j.ijbiomac.2017.11.097, PMID: 29155200

[19] Mujtaba M Ali Q Yilmaz BA Seckin Kurubas M Ustun H Erkan M. Understanding the effects of chitosan, chia mucilage, levan based composite coatings on the shelf life of sweet cherry. Food Chem. (2023) 416:135816. doi: 10.1016/j.foodchem.2023.135816 36893634

[20] Certel M Uslu MK Ozdemir F. Effects of sodium caseinate- and milk protein concentrate-based edible coatings on the postharvest quality of Bing cherries. J Sci Food Agric. (2004) 84:1229–34. doi: 10.1002/jsfa.1755

[21] Tokatlı K Demirdöven A. Effects of chitosan edible film coatings on the physicochemical and microbiological qualities of sweet cherry (*Prunus avium* L.). Sci Hortic. (2020) 259:108656. doi: 10.1016/j.scienta.2019.108656

[22] Yaseen D Sabbah M Al-Asmar A Altamimi M Famiglietti M Giosafatto CVL. Functionality of films from *Nigella sativa* defatted seed cake proteins plasticized with grape juice: use in wrapping sweet cherries. Coatings. (2021) 11:1383. doi: 10.3390/coatings11111383

[23] Sajimon A Edakkadan AS Subhash AJ Ramya M. Incorporating oregano (*Origanum vulgare* L.) essential oil onto whey protein concentrate based edible film towards sustainable active packaging. J Food Sci Technol-Mysore. (2023) 60:2408–22. doi: 10.1007/s13197-023-05763-7, PMID: 37424588 PMC10326189

[24] Zhang Y-L Cui Q-L Wang Y Shi F Fan H Zhang Y-Q. Effect of edible carboxymethyl chitosan-gelatin based coating on the quality and nutritional properties of different sweet cherry cultivars during postharvest storage. Coatings. (2021) 11:396. doi: 10.3390/coatings11040396

[25] Aday MS Caner C. Understanding the effects of various edible coatings on the storability of fresh cherry. Packag Technol Sci. (2010) 23:441–56. doi: 10.1002/pts.910

[26] Yuan Y Tian H Huang R Liu H Wu H Guo G. Fabrication and characterization of natural polyphenol and ZnO nanoparticles loaded protein-based biopolymer multifunction electrospun nanofiber films, and application in fruit preservation. Food Chem. (2023) 418:135851. doi: 10.1016/j.foodchem.2023.135851 36944306

[27] Das D Mir NA Chandla NK Singh S. Combined effect of pH treatment and the extraction pH on the physicochemical, functional and rheological characteristics of amaranth (*Amaranthus hypochondriacus*) seed protein isolates. Food Chem. (2021) 353:129466. doi: 10.1016/j.foodchem.2021.129466, PMID: 33735770

[28] Montes-de-Oca-Ávalos JM Altamura D Herrera ML Huck-Iriart C Scattarella F Siliqi D. Physical and structural properties of whey protein concentrate – corn oil – TiO2 nanocomposite films for edible food-packaging. Food Packag Shelf Life. (2020) 26:100590. doi: 10.1016/j.fpsl.2020.100590

[29] Zhang Z Huang X Li S Zhang C Luo K. Preparation and characterization of zein-sulfated cardamine hupingshanensis polysaccharide composite films. Food Sci Nutr. (2021) 9:6737–45. doi: 10.1002/fsn3.2625, PMID: 34925803 PMC8645725

[30] Maryam Adilah ZA Nur Hanani ZA. Storage stability of soy protein isolate films incorporated with mango kernel extract at different temperature. Food Hydrocoll. (2019) 87:541–9. doi: 10.1016/j.foodhyd.2018.08.038

[31] Moghadam M Salami M Mohammadian M Khodadadi M Emam-Djomeh Z. Development of antioxidant edible films based on mung bean protein enriched with pomegranate peel. Food Hydrocoll. (2020) 104:105735. doi: 10.1016/j.foodhyd.2020.105735

[32] Zhang Y Jing X Chen Z Wang X. Purification and identification of antioxidant peptides from millet gliadin treated with high hydrostatic pressure. LWT. (2022) 164:113654. doi: 10.1016/j.lwt.2022.113654

[33] Basiak E Geyer M Debeaufort F Lenart A Linke M. Relevance of interactions between starch-based coatings and plum fruit surfaces: a physical-chemical analysis. Int J Mol Sci. (2019) 20:2220. doi: 10.3390/ijms20092220, PMID: 31064114 PMC6539741

[34] Colussi R Ferreira da Silva WM Biduski B Mello El Halal SL da Rosa Zavareze E Guerra Dias AR. Postharvest quality and antioxidant activity extension of strawberry fruit using allyl isothiocyanate encapsulated by electrospun zein ultrafine fibers. LWT. (2021) 143:111087. doi: 10.1016/j.lwt.2021.111087

[35] Gharibzahedi SMT Ahmadigol A Khubber S Altintas Z. Whey protein isolate/jujube polysaccharide-based edible nanocomposite films reinforced with starch nanocrystals for the shelf-life extension of banana: optimization and characterization. Int J Biol Macromol. (2022) 222:1063–77. doi: 10.1016/j.ijbiomac.2022.09.232, PMID: 36181883

[36] Muley AB Singhal RS. Extension of postharvest shelf life of strawberries (Fragaria ananassa) using a coating of chitosan-whey protein isolate conjugate. Food Chem. (2020) 329:127213. doi: 10.1016/j.foodchem.2020.127213 32516713

[37] Bishnoi S Trifol J Moriana R Mendes AC. Adjustable polysaccharides-proteins films made of aqueous wheat proteins and alginate solutions. Food Chem. (2022) 391:133196. doi: 10.1016/j.foodchem.2022.133196, PMID: 35609460

[38] Sood A Saini CS. Red pomelo peel pectin based edible composite films: effect of pectin incorporation on mechanical, structural, morphological and thermal properties of composite films. Food Hydrocoll. (2022) 123:107135. doi: 10.1016/j.foodhyd.2021.107135

[39] Samsi MS Kamari A Din SM Lazar G. Synthesis, characterization and application of gelatin–carboxymethyl cellulose blend films for preservation of cherry tomatoes and grapes. J Food Sci Technol. (2019) 56:3099–108. doi: 10.1007/s13197-019-03809-3, PMID: 31205364 PMC6542916

[40] Zhang W Rhim J-W. Titanium dioxide (TiO2) for the manufacture of multifunctional active food packaging films. Food Packag Shelf Life. (2022) 31:100806. doi: 10.1016/j.fpsl.2021.100806

[41] Garrido T Etxabide A Guerrero P de la Caba K. Characterization of agar/soy protein biocomposite films: effect of agar on the extruded pellets and compression moulded films. Carbohydr Polym. (2016) 151:408–16. doi: 10.1016/j.carbpol.2016.05.089 27474583

[42] Kchaou H Jridi M Abdelhedi O Nasreddine B Karbowiak T Nasri M. Development and characterization of cuttlefish (*Sepia officinalis*) skin gelatin-protein isolate blend films. Int J Biol Macromol. (2017) 105:1491–500. doi: 10.1016/j.ijbiomac.2017.06.056, PMID: 28619638

[43] Chen W Ding J Yan X Yan W He M Yin G. Plasticization of cottonseed protein/polyvinyl alcohol blend films. Polymers. (2019) 11:2096. doi: 10.3390/polym11122096 31847379 PMC6960829

[44] Acquah C Zhang Y Dubé MA Udenigwe CC. Formation and characterization of protein-based films from yellow pea (*Pisum sativum*) protein isolate and concentrate for edible applications. Curr Res Food Sci. (2020) 2:61–9. doi: 10.1016/j.crfs.2019.11.008, PMID: 32914112 PMC7473362

[45] Gagliarini N Figoli CB Piermaria J Bosch A Abraham AG. Unraveling molecular interactions in whey protein-kefiran composite films to understand their physicochemical and mechanical properties. Food Biosci. (2022) 50:102012. doi: 10.1016/j.fbio.2022.102012

[46] Lin L Mei C Shi C Li C Abdel-Samie MA Cui H. Preparation and characterization of gelatin active packaging film loaded with eugenol nanoparticles and its application in chicken preservation. Food Biosci. (2023) 53:102778. doi: 10.1016/j.fbio.2023.102778

[47] Hu Y Shi L Ren Z Hao G Chen J Weng W. Characterization of emulsion films prepared from soy protein isolate at different preheating temperatures. J Food Eng. (2021) 309:110697. doi: 10.1016/j.jfoodeng.2021.110697

[48] Zhang C Wang Z Li Y Yang Y Ju X He R. The preparation and physiochemical characterization of rapeseed protein hydrolysate-chitosan composite films. Food Chem. (2019) 272:694–701. doi: 10.1016/j.foodchem.2018.08.097, PMID: 30309600

[49] Mir NA Riar CS Singh S. Effect of film forming solution pH on antibacterial, antioxidant and structural characteristics of edible films from modified quinoa protein. Food Hydrocoll. (2023) 135:108190. doi: 10.1016/j.foodhyd.2022.108190

[50] Sarıcaoğlu FT Turhan S. Physical, chemical, thermal and microstructural characterization of edible films from mechanically deboned chicken meat proteins. J Polym Environ. (2019) 27:1071–85. doi: 10.1007/s10924-019-01410-5

[51] Yuan G Jia Y Pan Y Li W Wang C Xu L. Preparation and characterization of shrimp shell waste protein-based films modified with oolong tea, corn silk and black soybean seed coat extracts. Polym Test. (2020) 81:106235. doi: 10.1016/j.polymertesting.2019.106235

[52] He M Zhang B Dou Y Yin G Cui Y. Blend modification of feather keratin-based films using sodium alginate. J Appl Polym Sci. (2017) 134:44680. doi: 10.1002/app.44680

[53] Kumari N Bangar SP Petrů M Ilyas RA Singh A Kumar P. Development and characterization of fenugreek protein-based edible film. Foods. (2021) 10:1976. doi: 10.3390/foods10091976, PMID: 34574085 PMC8465570

[54] Yu Y-P Lai S-J Chang C-R Chen W-C Wu S-H Lu C-P. Peptidomic analysis of low molecular weight antioxidative peptides prepared by lotus (*Nelumbo nucifera* Gaertn.) seed protein hydrolysates. LWT-Food Sci Technol. (2021) 144:111138. doi: 10.1016/j.lwt.2021.111138

[55] Lu X Zhang L Sun Q Song G Huang J. Extraction, identification and structure-activity relationship of antioxidant peptides from sesame (*Sesamum indicum* L.) protein hydrolysate. Food Res Int. (2019) 116:707–16. doi: 10.1016/j.foodres.2018.09.001 30716998

[56] Xue H-Y Zhao Y Liu Z-H Wang X-W Zhang J-W Peng X. Recovery of yam soluble protein from yam starch processing wastewater. Sci Rep. (2020) 10:5384. doi: 10.1038/s41598-020-62372-6 32214175 PMC7096408

[57] Aguirre-Joya JA Ventura-Sobrevilla J Martínez-Vazquez G Ruelas-Chacón X Rojas R Rodríguez-Herrera R. Effects of a natural bioactive coating on the quality and shelf life prolongation at different storage conditions of avocado (*Persea americana* mill.) cv. Hass Food Packag Shelf Life. (2017) 14:102–7. doi: 10.1016/j.fpsl.2017.09.003

[58] Xiao Y Liu Y Kang S Xu H. Insight into the formation mechanism of soy protein isolate films improved by cellulose nanocrystals. Food Chem. (2021) 359:129971. doi: 10.1016/j.foodchem.2021.129971 33962191

[59] Costa BP Carpiné D Ikeda M Pazzini IAE da Silva Bambirra Alves FE de Melo AM. Bioactive coatings from non-conventional loquat (*Eriobotrya japonica* Lindl.) seed starch to extend strawberries shelf-life: an antioxidant packaging. Prog Org Coat. (2023) 175:107320. doi: 10.1016/j.porgcoat.2022.107320

[60] Nandane AS Dave RK Rao TVR. Optimization of edible coating formulations for improving postharvest quality and shelf life of pear fruit using response surface methodology. J Food Sci Technol. (2017) 54:1–8. doi: 10.1007/s13197-016-2359-9, PMID: 28242897 PMC5305695

[61] Soradech S Nunthanid J Limmatvapirat S Luangtana-anan M. Utilization of shellac and gelatin composite film for coating to extend the shelf life of banana. Food Control. (2017) 73:1310–7. doi: 10.1016/j.foodcont.2016.10.059

[62] Mei S Fu B Su X Chen H Lin H Zheng Z. Developing silk sericin-based and carbon dots reinforced bio-nanocomposite films and potential application to litchi fruit. LWT. (2022) 164:113630. doi: 10.1016/j.lwt.2022.113630

[63] Yousuf B Srivastava AK Ahmad S. Application of natural fruit extract and hydrocolloid-based coating to retain quality of fresh-cut melon. J Food Sci Technol. (2020) 57:3647–58. doi: 10.1007/s13197-020-04397-3, PMID: 32903859 PMC7447745

